# Androgen receptor suppresses lung cancer invasion and increases cisplatin response *via* decreasing TPD52 expression

**DOI:** 10.7150/ijbs.84577

**Published:** 2023-07-16

**Authors:** Shiqing Liu, Chengping Hu, Min Li, Wolong Zhou, Ronghao Wang, Yao Xiao

**Affiliations:** 1 Department of Respiratory Medicine, Xiangya Hospital, Central South University, Changsha, 410008, China.; 2 Department of Biochemistry and Molecular Biology, School of Basic Medical Sciences, Southwest Medical University, Luzhou 646000, China.; 3 Xiangya Lung Cancer Center, Xiangya Hospital, Central South University, Changsha, 410008, China.; 4 National Clinical Research Center for Geriatric Disorders, Xiangya Hospital, Central South University, Changsha, 410008, China.; 5 Department of Thoracic Surgery, Xiangya Hospital, Central South University, Changsha, 410008, China.; 6 Department of General Surgery, Xiangya Hospital, Central South University, Changsha, 410008, China.; 7 International Joint Research Center of Minimally Invasive Endoscopic Technology Equipment & Standards, Changsha 410008, China.

**Keywords:** lung cancer, androgen receptor, invasion, cisplatin response, TPD52

## Abstract

Lung cancer, as the most commonly diagnosed malignancy, still accounts for the leading cause of cancer-related deaths worldwide. The high rate of mortality and tumor recurrence has prompted clinicians and scientists to urgently explore new targets for improved treatment. Previous studies have indicated a potential role of the androgen receptor (AR) in the progression of non-small cell lung cancer (NSCLC). However, the precise mechanisms underlying this association, particularly its relation to TPD52-mediated cell invasion and cisplatin (DDP) response, have not been fully elucidated. Therefore, further investigation is necessary to gain a better understanding of these mechanisms and their potential implications for lung cancer treatment. In this study, we discovered that AR can suppress NSCLC cell invasion and increase cisplatin response by downregulating the expression of circular RNA (circRNA), specifically circ-SLCO1B7. This suppression is achieved through the direct binding of AR to the 5' promoter region of the host gene SLCO1B7. The decreased expression of circ-SLCO1B7, mediated by AR, released miR-139-5p back to the RISC (RNA induced silencing complex), where it bonds to the 3' untranslated region (3'UTR) of Tumor Protein D52 (TPD52) messenger RNA, resulting in TPD52 reduction. The *in vivo* data also validated the functional contribution of AR/circ-SLCO1B7/miR-139-5p/TPD52 axis to lung cancer progression. Furthermore, analysis of human NSCLC databases and clinical specimens confirmed the association of the AR/circ-SLCO1B7/miR-139-5p/TPD52 signaling pathway with NSCLC progression. Collectively, the results from our study suggest that AR can suppress lung cancer cell invasion and increase DDP response by modulating the circ-SLCO1B7/miR-139-5p/TPD52 signaling pathway. Targeting this novel signaling pathway may be a new therapeutic strategy to effectively constrain NSCLC development.

## Introduction

As the leading cause of cancer related deaths (18.0% of the total cancer deaths) in the world, lung cancer ranks the second commonly diagnosed cancer (11.4% of total cases) 1. Lung cancer has the high incidence and mortality in both men and women. However, data also indicate that gender disparities are observed in the incidence and prognosis of lung cancer 2-4. Specifically, a survey has shown that women who have never smoked are approximately 2.5 times more prone to developing lung cancer compared to men 5, 6. It has also been observed that men tend to receive a lung cancer diagnosis at an older age compared to women 7. These observations suggest a potential role of sex hormones and their receptors in the initiation and progression of lung cancer 6, 8. More significantly, clinical evidence illustrated that AR was down-regulated in NSCLC tissues, and NSCLC patients with a high level of AR had a good survival rate 9.

As a sex hormone receptor, AR is a transcription factor for the regulation of a variety of genes upon androgen binding. Generally, AR belongs to the nuclear receptor subfamily, which encompasses other receptors such as estrogen receptor (ER), progesterone receptor (PR), and glucocorticoid receptor (GR) 10. The AR gene is located on the X chromosome at position q11-12 and consists of eight exons, encoding a protein with four functional domains 11. It is gradually acknowledged that AR plays various roles in the development of prostate cancer, bladder cancer, kidney cancer, liver cancer, etc^12^-^17^. Nevertheless, the detailed role of AR in NSCLC progression is still elusive and requires extensive investigation.

Circular RNA (circRNA) was initially identified in RNA viruses and was considered an abnormal product of mRNA splicing 18. However, it has now been recognized that circRNAs are widespread in human cells and play crucial roles in post-transcriptional gene regulation 19, 20. Unlike linear RNAs that possess distinct 5' and 3' ends, circRNAs form a closed loop structure that confers resistance to RNA exonucleases and stablizes their integrity 21. Emerging evidence suggest that circRNAs mainly function as "sponges" for microRNAs (miRNAs), thereby modulating gene expression and contributing to various biological processes and cancer development 15, 22. Although the involvement of circRNAs in lung cancer development has been documented 8, 23, 24, much more efforts should be made to dissect the roles of circRNAs in this type of disease.

MicroRNAs (miRNAs) represent a class of non-coding RNAs characterized by their short length, typically ranging from 18 to 25 nucleotides. These small RNA molecules have been extensively studied and shown to modulate mRNA stability and translation, primarily by binding to the 3' untranslated regions (3'UTRs) of target genes 17, 25, 26. MiRNAs have been implicated in the regulation of various cancer types, including prostate cancer, lung cancer, liver cancer, and others 27-29.

Tumor Protein D52, TPD52 in short, enables the progression of breast cancer 30. In lung squamous cell carcinoma, TPD52 is predisposed to promote cancer cell aggressiveness 31. These findings suggest that TPD52 may hold promise as a potential therapeutic target for the treatment of various human cancers.

In this study, circ-SLCO1B7, identified as the downstream target of AR, plays a role in NSCLC invasion and cisplatin response. Specifically, circ-SLCO1B7 functions as a sponge of miR-139-5p, thereby releasing its negative regulation on TPD52 and promoting NSCLC progression. This newly discovered mechanism highlights the involvement of AR in modulating the circ-SLCO1B7/miR-139-5p/TPD52 signaling pathway, which impacts the progression of NSCLC cells.

## Materials and Methods

### Human tissues

Clinical samples were obtained from our hospital. The collection of these samples was conducted solely for research purposes. Informed consent was obtained from all patients prior to the study, indicating their willingness to contribute their tissue samples for scientific investigation. The collection and utilization of these samples were conducted in accordance with ethical guidelines and regulations to ensure the protection of patient rights and privacy. Ethical considerations were strictly adhered to during the study.

### Reagents and Materials

Normal rabbit IgG used in the study were procured from Santa Cruz. For Western blot analysis, anti-mouse or anti-rabbit secondary antibodies were obtained from Invitrogen.

### Cell culture and stable cell lines

A549 and H1299 cell lines were obtained from ATCC and cultured in 1640 medium (Invitrogen) supplemented with 10% FBS. The cells were maintained in a 5% CO_2_ humidified incubator at 37°C. Regular testing for bacterial and mycoplasma contamination, following ATCC's instructions, was performed every three months to ensure the cell lines' integrity and freedom from contamination.

### Plasmids construction

The functional plasmid, as well as the psPAX2 (10 μg) and pMD2.G (10 μg), were introduced into 293T cells using the calcium phosphate transfection method. Following a 48-hour incubation period, the lentivirus supernatant was harvested. The concentrated lentivirus was either used immediately or stored at -80°C for future use. To select for shRNA-infected cells, a concentration of 5µg/ml puromycin was utilized.

### MTT assay

Cells were seeded at a density of 3000 cells per well in 96-well tissue culture plates and treated with or without cisplatin. After 48 hours, methylthiazolyldiphenyl-tetrazolium bromide (MTT) reagent was added to each well and incubated for 2 hours at 37°C. The formazan precipitate was dissolved in dimethyl sulfoxide (DMSO), and the absorbance was measured at 450 nm. Each sample was assayed in triplicate to ensure reliable results. This assay provided quantitative assessment of cell viability and proliferation in response to cisplatin treatment.

### Transwell invasion assay

The invasion assay was performed using 8 µm transwell chambers (Corning Life Science) placed in 24-well plates. Initially, the upper chamber was coated with a 1:20 dilution of Matrigel (BD Biosciences) in a volume of 100 µl per well. The plates were then incubated at 37 °C in a humidified incubator for 2-4 hours to allow for gel formation. Subsequently, a cell suspension containing 2-5×10^4^ cells in serum-free medium was added to the upper chamber of a Transwell insert, while 750 μl of medium containing 10% FBS was added to the lower chamber. After incubating for 24 hours, the non-invaded cells in the upper chamber were removed by washing. The invaded cells that passed through the insert and adhered to the lower surface were fixed with methanol and stained with a 0.1% (w/v) solution of crystal violet. To ensure accuracy and consistency, three wells were used to measure the invaded cells of each group, and the experiment was conducted at least three times.

### Western blot assay

Cellular proteins were extracted by lysing the cells using RIPA buffer. A total of 30 µg of protein was loaded onto a polyacrylamide gel for SDS/PAGE electrophoresis. Following electrophoresis, the proteins were transferred from the gel to a polyvinylidene difluoride membrane (Millipore). The membrane was then blocked with a suitable blocking agent for 1 hour and washed with Tris-buffered saline with Tween (TBST) three times. Subsequently, the membrane was incubated overnight at 4°C with the appropriate primary antibody in a cold room to facilitate specific protein detection. Afterward, the membrane was washed with TBST three times and incubated with secondary antibodies conjugated to a detection system. Finally, the protein signals on the membrane were visualized using an ECL system (Thermo Fisher Scientific). The primary antibodies used in this study are: AR (5153, CST), NcoR1 (34271, CST), TPD52 (sc-166732, Santa Cruz), GAPDH (sc-47724, Santa Cruz).

### qRT-PCR assay

Total RNA from lung cancer cells was extracted using Trizol reagent (Invitrogen), and 2 μg of total RNA was used for reverse transcription. The expression of RNA (mRNA and miRNA) was analyzed and quantified using the Bio-Rad CFX96 system. The results were normalized using GAPDH as the reference gene for mRNA or U6 for miRNA, and the relative expression levels were determined using the 2ΔΔCt method. All primers used in the study were obtained from IDT Company. Targeted primer sequences were listed in **supplementary Table 1**.

### Immunoprecipitation assay (IP)

Cell lysates were precleared by protein A-agarose for 2 hours and incubated with anti-AR antibody (2.0 μg) or equal amount of rabbit IgG (sc-2027, Santa Cruz) overnight at 4°C. Next day, protein A-agarose beads blocked with 5% BSA were utilized to pull down antibody-AR complex. After extensive wash with cell lysis buffer, 1XSDS loading buffer was added to the agarose beads for western blotting analysis.

### Chromatin immunoprecipitation assay (ChIP)

To preclear the cell lysates, a sequential treatment with normal rabbit IgG (sc-2027, Santa Cruz) and protein A-agarose was performed. Subsequently, the cell lysate was incubated with anti-AR antibody (2.0 μg) overnight at 4°C. As a negative control, IgG was included in the reaction. Primer sets were specifically designed to amplify a target sequence within the human SLCO1B7 promoter region. The PCR products were then separated and visualized using agarose gel electrophoresis.

### Luciferase reporter assay

The 5'-promoter region of the human SLCO1B7 gene was cloned into the pGL3-basic luciferase reporter vector (Promega) to generate a reporter construct. This construct allows for the study of transcriptional activity associated with the SLCO1B7 promoter. To investigate the specific role of the androgen receptor (AR) binding site within the SLCO1B7 promoter, a mutant AR binding site was created using Quick Change mutagenesis. The TPD52 3' UTR fragment containing wild-type or mutant miRNA-response elements was cloned downstream of the Renilla luciferase ORF in the psiCHECK-2 vector (Promega). Lung cancer cells with appropriate density in 24-well plates were transfected with luciferase reporter plasmids (100 ng/well) using Lipofectamine 3000 transfection reagent (Invitrogen, Carlsbad, CA) following the manufacturer's instructions. PRL-TK (10 ng/well) was a transfection internal control. Luciferase activity was measured after 36-48 hours.

### *In vivo* studies

A total of 1x10^6^ cells were suspended in a mixture of 50 μL serum-free media and 50 μL Matrigel (Becton Dickinson & Co). The cell mixture was injected into the left lateral thorax of mice, following a previously described method 32, 33. IVIS was used to detected tumor growth and metastasis weekly. After 8 weeks, the mice were sacrificed, and tumors as well as any metastases were collected for further studies. All animal experiments were conducted in accordance with the guidelines and regulations of the institutional animal care committee at our university.

### Statistical Analysis

All statistical analyses were conducted using SPSS 28.0 software. All values are presented as mean ±SD, and statistical significance was determined using the t-test. Differences among groups were analyzed using the one-way ANOVA test, with *p < 0.05, **p < 0.01, ***p < 0.001 indicating significance, and "ns" representing non-significance.

### Data Availability Statement

The data supporting the findings of this study are available within the article.

## Results

### AR suppresses lung cancer cell invasion and increases cisplatin response

**Figure 1:**
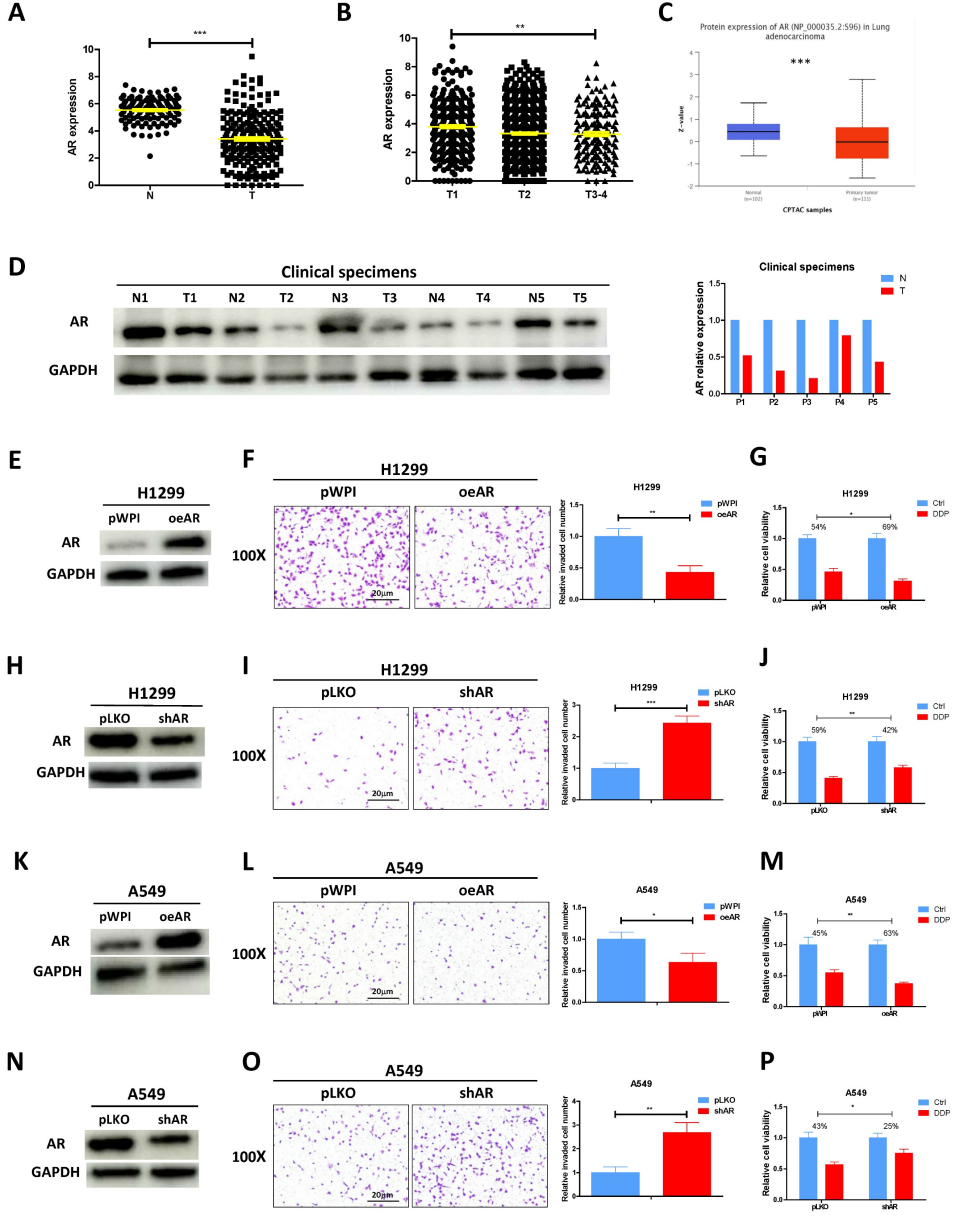
** AR suppresses lung cancer cell invasion and increases cisplatin response. A.** Analysis of lung cancer patient data from the TCGA database revealed differential expression of AR in normal (N) tissues and tumor (T) tissues. **B.** AR expression was evaluated in different grades (T1-4) of lung cancer patients from the TCGA database. **C.** Analysis of AR protein level in normal tissues and lung tumor tissues according to CPTCA database. **D.** AR protein expressions were detected using western blot analysis in five pairs of clinical samples. GAPDH was an internal control. **E, H, K, N.** Western blot analysis was performed to verify the efficiency of overexpression (oeAR) or knockdown (shAR) of AR in H1299 and A549 cells.** F, I, L, O.** Chamber-transwell invasion assays were conducted to measure the invasion of H1299 and A549 cells transfected with oeAR or shAR. The number of invaded cells was quantified in five randomly chosen microscopic fields (×100) in each experiment and averaged. Left, representative images of invaded cells. Right, statistical analysis. **G, J, M, P.** MTT assays were carried out to determine the response to DDP (cisplatin) in H1299 and A549 cells transfected with oeAR or shAR. All quantitations are reported as mean ± standard deviation (SD). Statistical analysis was performed using the t-test, and the significance levels are denoted as *p < 0.05, **p < 0.01, and ***p < 0.001.

According to the analysis of TCGA database, AR mRNA level was found to be decreased in lung cancer tumor tissues as compared to the normal tissues **(Fig. 1A)**. Furthermore, a correlation was observed between the stage of lung cancer and the level of AR, with higher tumor stages showing lower AR expression levels** (Fig. 1B)**. CPTAC database also revealed that lung cancer samples selected to express AR protein at a relatively low level (**Fig. 1C**). Result from the western blotting of AR in our collected clinical samples also demonstrated it was evidently decreased in lung cancer tumor tissues as compared to adjacent normal tissues **(Fig. 1D),** highly corroborating with the online analysis. To investigate the functional role of AR in lung cancer, we first efficiently overexpressed AR (oeAR) into H1299 and A549 cells **(Fig. 1E and K)**. As expected, oeAR exerted a suppressive effect on lung cancer cell invasion and increased cisplatin response of H1299 and A549 cells **(Fig. 1F, G, L, M)**. Conversely, AR reduction, achieved by introducing AR-shRNA (shAR) **(Fig. 1H and N),** resulted in a significant addition in lung cancer cell invasion and decreased cisplatin response of H1299 and A549 cells **(Fig. 1I, J, O, P)**.

### AR suppresses lung cancer cell progression through circ-SLCO1B7

**Figure 2:**
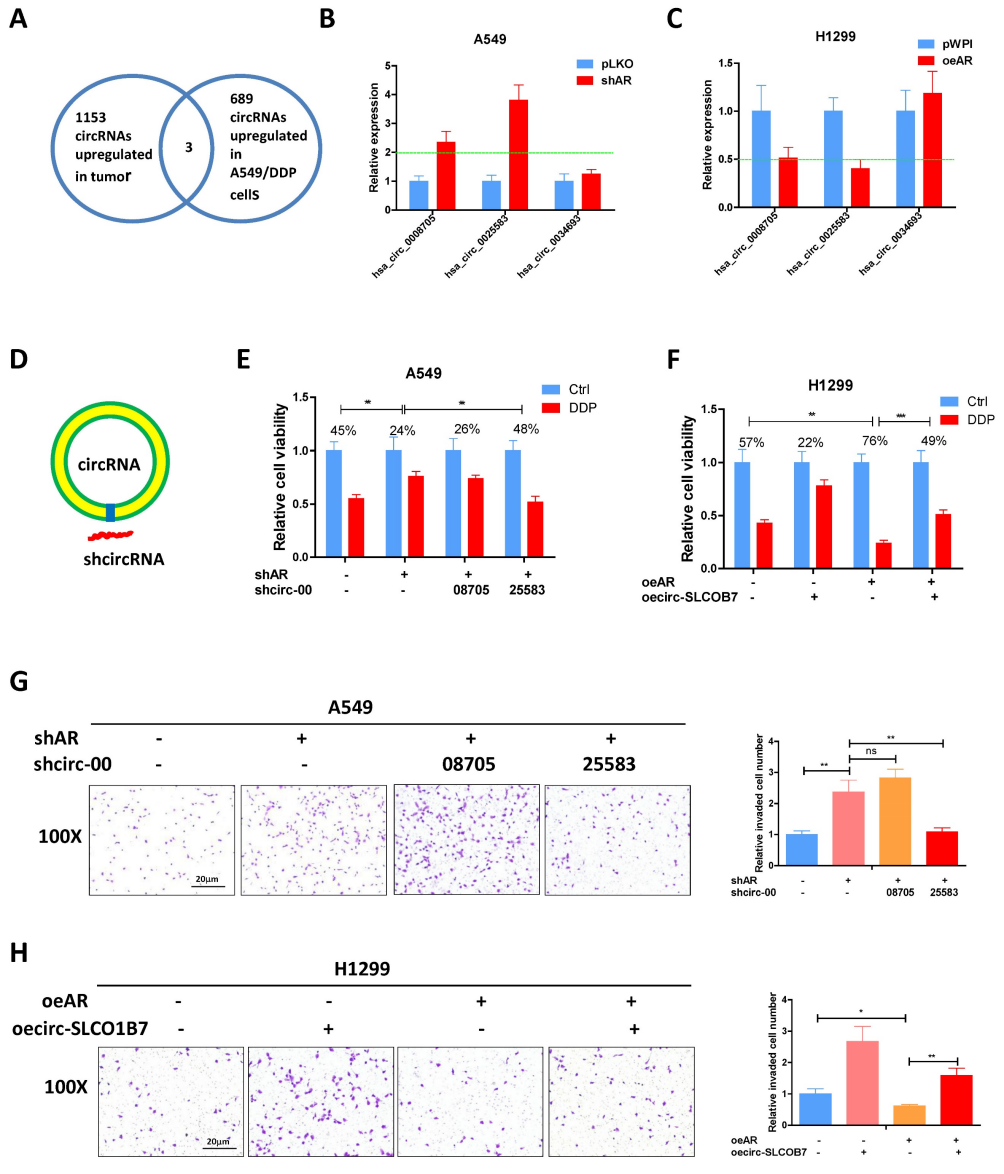
** AR suppresses lung cancer cell progression through circ-SLCO1B7. A.** The overlapping circRNAs between two studies were identified. **B-C.** qRT-PCR was performed to assess the expression levels of the three circRNAs in the shAR and pLKO A549 cells (**B**) and in the oeAR and pWPI H1299 cells (**C**). **D.** A schematic illustration was provided to demonstrate how to design the shRNA for circRNA knockdown (sh-circ). **E, F.** MTT assays were carried out to measure the DDP response of A549 cells transfected with shAR/shcircRNA and H1299 cells transfected with oeAR/oecirc-SLCO1B7. **G.** Chamber-transwell invasion assay was conducted to evaluate the invasion capacity of A549 cells after knockdown of circRNA and/or shAR. **H.** Chamber-transwell invasion assay was performed to assess the invasion capacity of H1299 cells after overexpression of AR and circ-SLCO1B7 (oecirc-SLCO1B7). All quantitations are presented as mean ±SD, and statistical significance was determined using the t-test, with *p < 0.05, **p < 0.01, ***p < 0.001 indicating significance, and "ns" representing non-significance.

Previous studies have provided evidence that AR can inhibit the progression of lung cancer cells 9. However, the relationship between AR and the biology of circRNA regarding to lung cancer cell invasion and cisplatin response remains unclear. Previously, Zhu X et al. conducted a microarray analysis to examine the circRNA profiles of 3 paired lung cancer samples 34 and identified 1153 up-regulated circRNAs in lung cancer tissues. In another study, Lu H et al. performed deep sequencing to characterize the circRNA profiles in A549 cells and the cisplatin-resistant counterparts, identifying 689 up-regulated circRNAs in the drug-resistant cells 35. To connect it, we focused on the three circRNAs which were consistently upregulated in lung cancer tissues and cisplatin resistant 549 cells **(Fig. 2A)** and tested whether AR had a regulation on them. The results demonstrated that hsa_circ_0008705 (circ-CABYR) and hsa_circ_0025583 (circ-SLCO1B7) consistently displayed changes in expression in response to AR manipulation **(Fig. 2B and C),** suggesting a potential regulatory role of AR in modulating the expression of these circRNAs in lung cancer cells.

To further investigate the potential role of these two circRNAs in regulating lung cancer cell progression, we designed specific shRNAs targeting the splice junctions of hsa_circ_0008705 and hsa_circ_0025583 (sh-circRNA) **(Fig. 2D)**. Using transwell assays with matrigel-coated filters, we assessed whether knockdown of hsa_circ_0008705 or hsa_circ_0025583 had any impacts on the cell invasion and cisplatin response of lung cancer cells. The results showed that only knockdown of hsa_circ_0025583, but not hsa_circ_0008705, was able to counteract shAR mediated response to cisplatin **(Fig. 2E)** and cell invasion induction **(Fig. 2G)** in A549 cells. Additionally, overexpression of circ-SLCO1B7 could attenuate oeAR mediated cisplatin response **(Fig. 2F)** and cell invasion **(Fig. 2H)** in H1299 cells and A549 cells (**Supplementary Fig. 1A, B**).

Taken together, these findings support the notion that AR functions as a tumor suppressor of lung cancer progression through the regulation of circ-SLCO1B7.

### Circ-SLCO1B7 enhances the invasive ability of lung cancer cell

**Figure 3:**
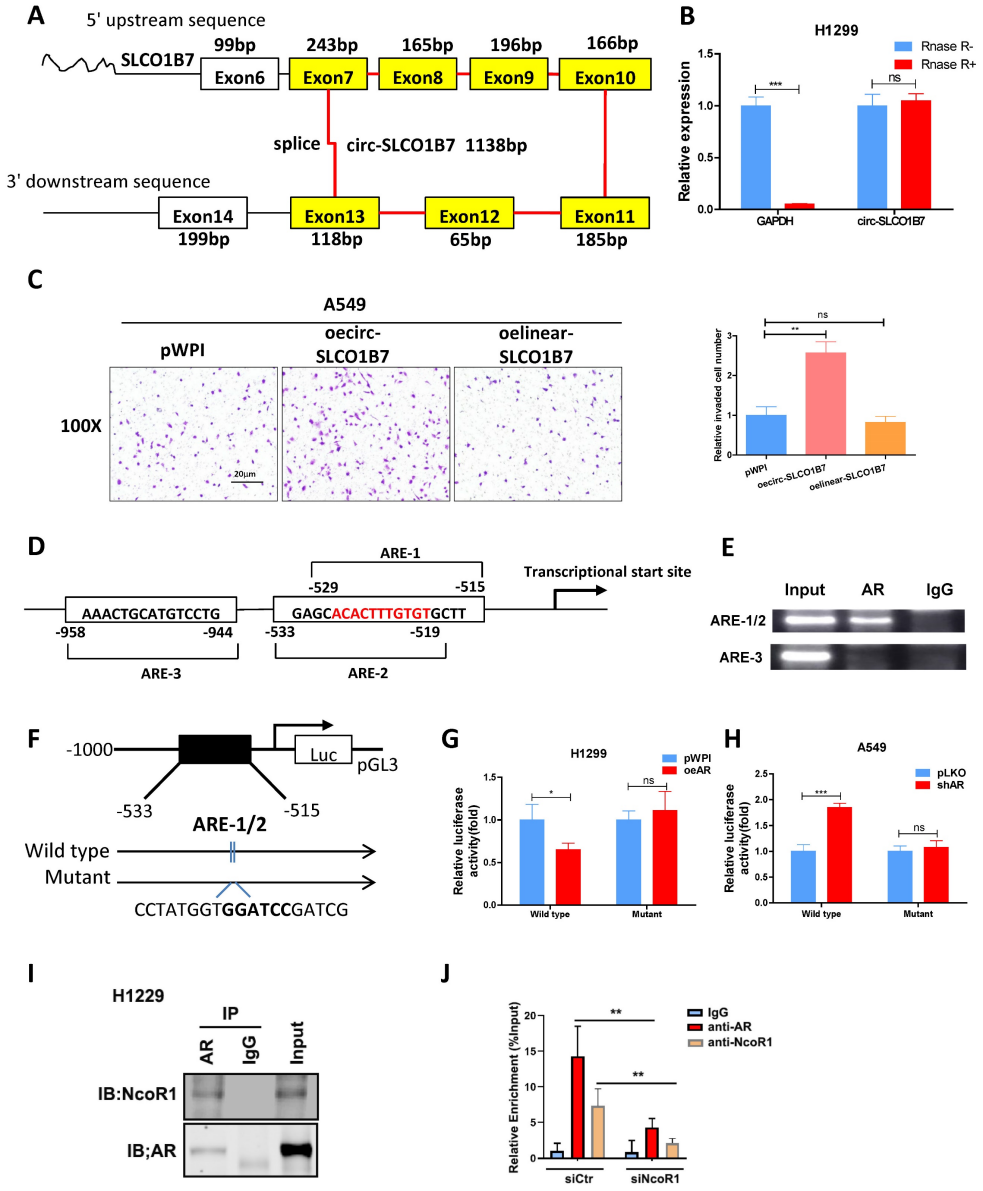
** Circ-SLCO1B7 enhances the invasive ability of lung cancer cell and AR transcriptionally regulates circ-SLCO1B7 expression. A.** The schematic diagram illustrates the genomic location and splicing pattern of circ-SLCO1B7. **B.** qRT-PCR was used to detect the expression of circ-SLCO1B7 and GAPDH mRNA in H1299 cells treated with or without RNase R. **C.** Chamber-transwell invasion assay was performed to assess the invasion capacity after the addition of circ-SLCO1B7 and linear-SLCO1B7 in A549 cells. **D.** Three potential androgen response elements (AREs) were predicted within the 2 kb 5'-promoter region of SLCO1B7. **E.** Chromatin immunoprecipitation (ChIP) binding assay was conducted on H1299 cells. **F.** Wild-type and mutant pGL3-SLCO1B7 promoter reporter constructs were used. **G-H.** Luciferase activity was measured after transfection of wild-type or mutant circRNA-SLCO1B7 promoter reporter constructs in H1299 cells transfected with oeAR or pWPI (**G**) and in A549 cells transfected with shAR or pLKO (**H**). **I.** CoIP assay to detect the interaction of AR and NcoR1. **J.** NcoR1 depletion attenuated the recruitment of AR and NcoR1 to the promoter region of circ-SLCO1B7. All quantitations are presented as mean ±SD, and statistical significance was determined using the t-test, with *p < 0.05, **p < 0.01, ***p < 0.001 indicating significance, and "ns" representing non-significance.

Our analysis revealed that circ-SLCO1B7 is derived from exon 7, 8, 9, 10, 11, 12, and 13 of the SLCO1B7 gene, forming a circular RNA molecule with a length of 1138bp **(Fig. 3A)**. To validate the circular nature of circ-SLCO1B7, we detected its level using divergent primers that span the splice junction, before and after RNase-R digestion of RNAs. The results demonstrated that circ-SLCO1B7 was resistant to RNase-R digestion, while the abundance of GAPDH mRNA was remarkably decreased upon RNase-R digestion **(Fig. 3B),** highly supporting the circular nature of circ-SLCO1B7. Next, we investigated the contribution of circ-SLCO1B7 to the cell invasion of lung cancer cells using transwell assay. Our findings illustrated that circ-SLCO1B7 overexpression resulted in a more profound increase in A549 cell invasion compared to the linear-SLCO1B7 and the vector control **(Fig. 3C)**. Circ-SLCO1B7 overexpression also increased the cell invasion of H1229 cells (**Fig. 2H**).

Taking together, we conclude that circ-SLCO1B7, as a circular RNA, enhances the cell invasion of lung cancer.

### AR transcriptionally regulates circ-SLCO1B7 expression

As a hormone receptor, AR controls the expression levels of a variety of genes at the transcription level. To investigate whether AR transcriptionally regulates circ-SLCO1B7 expression, we first screened SLCO1B7 gene promoter for potential androgen response elements (AREs) using the Ensembl website and JASPAR database. Within the 2kb region of the SLCO1B7 promoter, three putative AREs were identified **(Fig. 3D and Supplementary Fig. 1C, D)**. Subsequently, we conducted a ChIP assay to confirm the binding of AR to the putative AREs in lung cancer cells. The data revealed that AR indeed binds to the ARE1/2 elements within the SLCO1B7 promoter region **(Fig. 3E)**.

To explore whether AR recruitment influences the promoter activity of circ-SLCO1B7, we generated a luciferase reporter construct containing a 1kb 5' promoter region of SLCO1B7, including the identified ARE1/2, and a mutant version of ARE1/2** (Fig. 3F)**. Luciferase assays were subsequently performed to assess the effect of AR modulation on the luciferase activity of our constructs. Consistent with our hypothesis, overexpression of AR (oeAR) led to a significant luciferase activity reduction in construct with wild-type SLCO1B7 promoter, while no such effect was observed in construct with the mutant SLCO1B7 promoter lacking functional ARE1/2 **(Fig. 3G)**. Conversely, knockdown of AR (shAR) resulted in a significant luciferase activity increase, which was abolished when the SLCO1B7 promoter was mutated **(Fig. 3H)**. These evidence suggest that AR binds to the promoter region of circ-SCLO1B7 to inhibit its transcription, which may be explained by the recruitment of NcoR1 (**Fig. 3I**), a classical of AR corepressor. Indeed, NcoR1 depletion by siRNA attenuated AR enrichment in the ARE1/2 promoter region of SLCO1B7 (**Fig. 3J**).

Collectively, the findings presented in **Fig. 3D-J** provide evidence that AR exerts a transcriptional regulation on circ-SLCO1B7 expression. This regulation occurs through the binding of AR to the ARE1/2 elements located within the 5' promoter region of the SLCO1B7 gene.

### AR/circ-SLCO1B7 axis decreases lung cancer progression by releasing miR-139-5p

**Figure 4:**
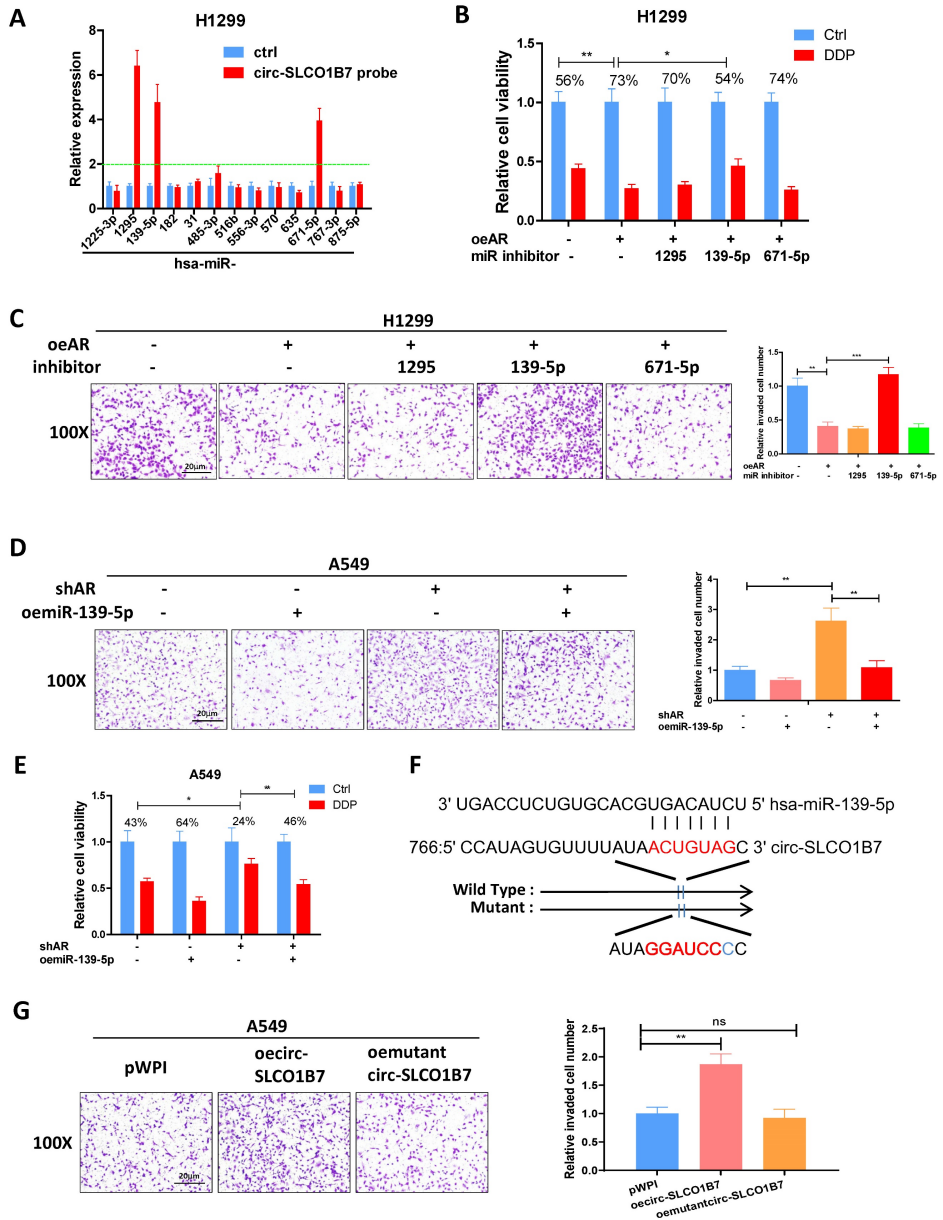
** AR/circ-SLCO1B7 axis decreases lung cancer progression by releasing miR-139-5p. A.** RNA pull-down assay was performed to determine the interaction of circ-SLCO1B7 with 13 potential candidate miRNAs using biotinylated oligonucleotides targeting the circular junction of circ-SLCO1B7. **B.** MTT assays were conducted to assess the DDP response of H1299 cells transfected with oeAR/miR inhibitor. **C.** Chamber-transwell invasion assay was used to evaluate the invasion capacity of H1299 cells transfected with oeAR/miR inhibitor. **D.** Chamber-transwell invasion assay was performed to assess the invasion capacity of A549 cells transfected with shAR or oemiR-139-5p. **E.** MTT assays were conducted to detect the DDP response of A549 cells transfected with shAR or oemiR-139-5p. **F.** Wild-type and mutant oecirc-SLCO1B7 constructs were used. **G.** Chamber-transwell invasion assays were performed to determine the efficiency of wild-type and mutant oecirc-SLCO1B7 in A549 cells. All quantitations are presented as mean ± SD, and statistical significance was calculated using the t-test. Significance levels are denoted as *p < 0.05, **p < 0.01, ***p < 0.001, and "ns" represents non-significance.

As circRNAs mainly functions as competing endogenous RNAs (ceRNAs) by sequestering miRNAs and thereby regulating the expression of target RNA transcripts 36, we performed an analysis using the Circular RNA Interactome database, which identified 13 miRNA candidates with high scores. To validate the interaction between circ-SLCO1B7 and these miRNAs, we conducted a RNA pull-down assay using a biotinylated oligonucleotide that is annealed to the circular junction of circ-SLCO1B7 (5'-TCCTGATAGTGCCCCAAATAT-3'). The results demonstrated that three miRNAs (miR-1295, miR-139-5p, and miR-671-5p) were enriched with circ-SLCO1B7, indicating a physical binding between these miRNAs and circ-SLCO1B7 **(Fig. 4A)**. However, only miR-139-5p inhibitor partially reversed the lung cancer suppressed cell progression and promoted cisplatin response by oeAR in H1299 cells **(Fig. 4B and C)**. Therefore, our focus was directed towards investigating the functional interaction between circ-SLCO1B7 and miR-139-5p. To further validate this interaction, a rescue assay was conducted in A549 cells, which revealed that the lung cancer progression and cisplatin response induced by shAR could be partially blocked by introducing miR-139-5p mimics **(Fig. 4D and E)**. Similar result was obtained from H1229 cells (**Supplementary Fig. 2A, B**).

To investigate the functional interaction between circ-SLCO1B7 and miR-139-5p, we generated a circ-SLCO1B7 construct with mutant miR-139-5p binding site** (Fig. 4F)**. Interestingly, when the mutant circ-SLCO1B7 was introduced into A549 cells, it failed to significantly promote cell invasion as compared to the wild type one **(Fig. 4G)**. We also cloned the linear sequence of wild type or mutated circ-SLCO1B7 into the downstream of luciferase within psiCHECK2 vector and performed the reporter assay to verify the direct interaction between circ-SLCO1B7 and miR-139-5p. As shown in **Supplementary Fig**. **2C**, miR-139-5p mimics could reduce the activity of wild type construct but had little impact on the mutated one. These findings suggest that circ-SLCO1B7 promotes lung cancer progression by acting as a sponge for miR-139-5p, thereby inhibiting its activity.

Overall, the results from **Fig. 4A-G** support the notion that the AR/circ-SLCO1B7 axis functions to titrate the biological effect of miR-139-5p, ultimately impacting lung cancer progression.

### AR/circ-SLCO1B7/miR-139-5p axis suppresses lung cancer cell progression *via* altering TPD52 expression

**Figure 5:**
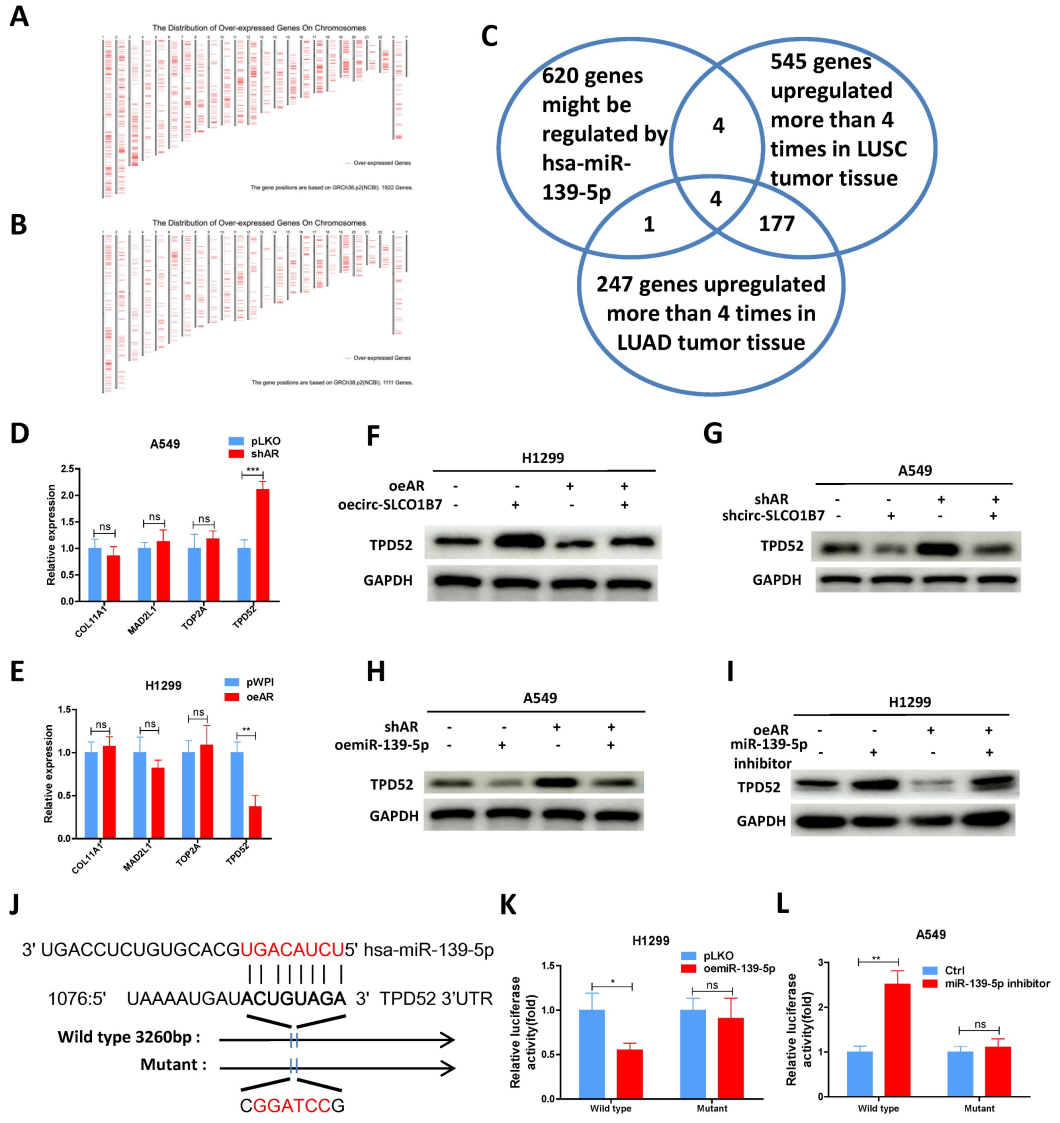
** AR/circ-SLCO1B7/miR-139-5p axis suppresses lung cancer cell progression *via* altering TPD52 expression. A.** The distribution of 1922 upregulated genes on chromosomes in LUSC. **B.** The distribution of 1111 upregulated genes on chromosomes in LUAD. **C.** Overlapping genes were identified among the three groups. **D-E.** qRT-PCR assay was performed to screen genes in A549 cells transfected with shAR compared to pLKO (**D**) and in H1299 cells transfected with oeAR compared to the vector pWPI (**E**). **F-I.** Western blot assays were conducted to detect TPD52 expression in the two cell lines by manipulating AR/circ-SLCO1B7 and AR/miR-139-5p. **J.** Sequence alignment of the TPD52 3'UTR with wild-type and mutant sequences. **K-L.** Luciferase reporter activity was measured after transfection of wild-type or mutant TPD52 3'UTR reporter constructs in H1299 cells with or without oemiR-139-5p (**K**) and in A549 cells treated with or without miR-139-5p inhibitor (**L**). All quantitations are presented as mean ± SD, and statistical significance was calculated using the t-test. Significance levels are denoted as *p < 0.05, **p < 0.01, ***p < 0.001, and "ns" represents non-significance.

To explore the downstream targets of miR-139-5p and their potential involvement in lung cancer progression, we utilized the miRDB database to identify 620 genes that could be potentially regulated by hsa-miR-139-5p. Additionally, we analyzed gene expression data from lung squamous cell carcinoma (LUSC) and lung adenocarcinoma (LUAD) tumor tissues using the GEPIA database, which revealed 1922 upregulated genes in LUSC and 1111 upregulated genes in LUAD **(Fig. 5A-B)**. Among these gene panels, we selected four candidates (COL11A1, MAD2L1, TOP2A, and TPD52) that were consistently identified by all three panels and exhibited more than a four-fold increase in expression **(Fig. 5C)**. Subsequently, we performed qRT-PCR analysis to assess the expression of these genes in two lung cancer cell lines after AR manipulation. The results demonstrated that only TPD52 level displayed a significant differential change upon shAR in A549 cells **(Fig. 5D)** and oeAR in H1299 cells **(Fig. 5E)**. Therefore, we decided to focus on TPD52 for further investigation regarding its potential links to AR, circ-SLCO1B7 and miR-139-5p in lung cancer progression.

To further investigate the relationship between AR, circ-SLCO1B7, miR-139-5p and TPD52 expression, we conducted several rescue assays. In H1299 cells, the addition of circ-SLCO1B7 increased TPD52 protein expression and partially reversed the suppressive effect of oeAR on TPD52 expression **(Fig. 5F)**. Conversely, in A549 cells, knockdown of circ-SLCO1B7 decreased TPD52 protein expression and effectively reversed the shAR-induced TPD52 protein level **(Fig. 5G)**. Of particular importance, we observed that the addition of miR-139-5p in A549 cells led to a decrease in TPD52 expression and effectively reversed the shAR-induced increase in TPD52 expression **(Fig. 5H)**. Conversely, treating H1299 cells with a miR-139-5p inhibitor resulted in an increase in TPD52 expression and partially reversed the inhibitory effect of AR on TPD52 expression **(Fig. 5I)**. We also found that the regulation of circ-SLCO1B7, miR-139-5p and TPD52 by AR was hormone dependent. As shown in **Supplementary Fig. 2D**, the expression levels of circ-SLCO1B7 and TPD52 were decreased while miR-139-5p level was increased, upon DHT treatment in A549 cells. Consistently, the expression levels of circ-SLCO1B7 and TPD52 were increased while miR-139-5p level was decreased when H1229 cells was exposed to enzalutamide (Enz) (**Supplementary Fig. 2E**).

To investigate the molecular mechanism by which miR-139-5p regulates TPD52 expression, we successfully identified a potential binding site located on the 3'UTR of TPD52 mRNA. To assess the functional significance of this interaction, we conducted a reporter assay using the psiCHECK2 vector carrying the wild-type and mutant miRNA-target sites **(Fig. 5J)**, Our results revealed that miR-139-5p significantly reduced the luciferase reporter activity of wild-type construct, while it had minimal impact on the luciferase activity of the mutated one **(Fig. 5K)**. Furthermore, the miR-139-5p inhibitor notably increased the luciferase activity of wild-type construct, while no such effect was observed in cells carrying the mutant 3'UTR of TPD52. **(Fig. 5L)**. Of note, TPD52 mRNA of miR-139-5p treated cells was degraded at much faster rate than that of control cells (**Supplementary Fig. 2F**). These results suggest that miR-139-5p directly targets the 3'UTR of TPD52 mRNA to suppress its protein expression.

In summary, the findings from **Fig. 5A-L** indicate that the AR/circ-SLCO1B7/miR-139-5p axis suppressed lung cancer cell progression by modulating TPD52 expression.

### Human clinical study to link the AR/ circ-SLCO1B7/ miR-139-5p/ TPD52 signaling to the lung cancer progression

**Figure 6:**
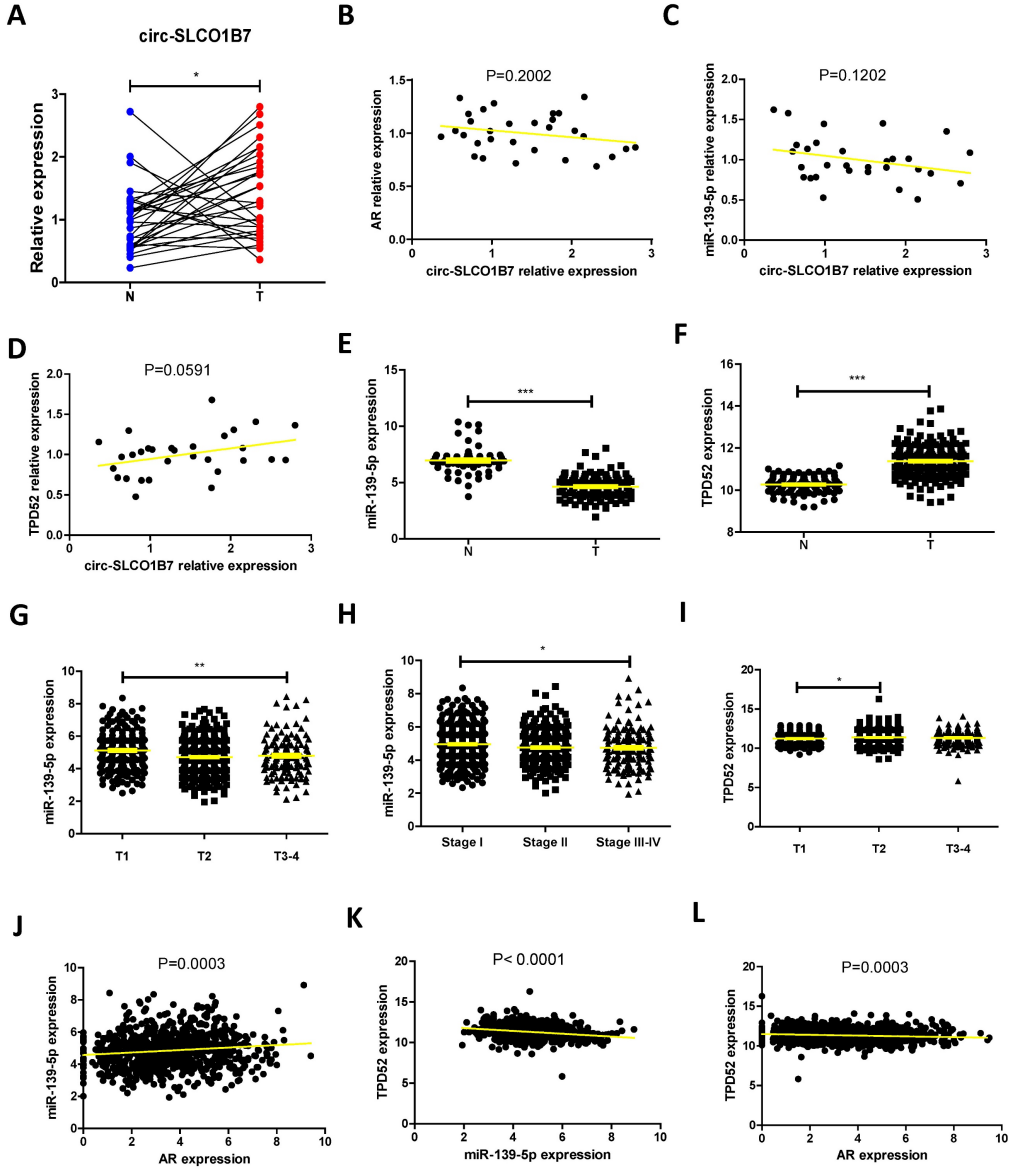
** Human clinical study to link the AR/ circ-SLCO1B7/ miR-139-5p/ TPD52 signaling to the lung cancer progression. A**.The expression level of circ-SLCO1B7 in our collected lung cancer tissues and the adjacent normal tissues. **B.** The correlation between circ-SLCO1B7 and AR of our collected lung cancer samples.** C.** The correlation between circ-SLCO1B7 and miR-139-5p of our collected lung cancer samples. **D.** The correlation between circ-SLCO1B7 and TPD52 of our collected lung cancer samples. **E-F**. Expression of miR-139-5p and TPD52 in normal (N) tissues and tumor (T) tissues according to the TCGA database. **G-H.** Expression of miR-139-5p in different grades (T1-4) and stages (Stage I-IV) of lung cancer. **I.** Expression of TPD52 in different grades (T1-4) of lung cancer. **J.** Correlation analysis of AR and miR-139-5p expression. **K.** Correlation analysis of miR-139-5p and TPD52 expression. **L.** Correlation analysis of AR and TPD52 expression. All quantitations are presented as mean ± SD, and p-values were calculated using the t-test. Significance levels are denoted as *p < 0.05, **p < 0.01, and ***p < 0.001.

To establish a clinical relevance of the in vitro findings, we collected clinical samples and data from lung cancer patients who had been underwent surgery at our hospital between 2020 and 2021. The analysis revealed significant correlations between expression of circ-SLCO1B7/ miR-139-5p and tumor size, T grade, N grade, and differentiation** (Table 1)**. The expression level of circ-SLCO1B7 was indeed increased in lung cancer samples as compared to the normal counterparts (**Fig. 6A**).

Further investigations illustrated that circ-SLCO1B7 was negatively correlated with AR and miR-139-5p but positively correlated TPD52 at expression level, although the trends were not reached a statistical significance probably owing to the limited sample size (**Fig. 6B, C, D**). We therefore analyzed their clinical values using TCGA data. First, we observed that lung cancer patients with lower TPD52 expression had higher survival rate **(Supplementary Fig. 3A)**. Relatively low levels of AR and miR-139-5p were observed in TPD52 high cohorts (**Supplementary Fig. 3B, C**). Moreover, miR-139-5p was expressed at higher level in normal tissues as compared to tumor tissues **(Fig. 6E)**, while TPD52 expression exhibited the opposite pattern, with higher expression in tumor tissues **(Fig. 6F)**. Additionally, miR-139-5p levels were gradually decreased as lung cancer progresses to high grade or stage **(Fig. 6G, H and Supplementary Fig. 3D)**, whereas TPD52 expression showed an opposite trend **(Fig. 6I)**. Furthermore, a positive correlation was observed between AR and miR-139-5p expression **(Fig. 6J)**, while miR-139-5p exhibited a negative correlation with TPD52 expression **(Fig. 6K)**, as well as AR and TPD52 expression **(Fig. 6L)**.

**Fig. 6A-L** and **Table 1** are in line with the findings from preclinical studies using multiple lung cancer cell lines. These results further support the notion that the AR/circ-SLCO1B7/miR-139-5p/TPD52 signaling pathway may have significant roles in the modulation of lung cancer progression.

### To investigate the role of the AR/circ-SLCO1B7/miR-139-5p/TPD52 signaling pathway in lung cancer progression in the* in vivo* mouse model

**Figure 7:**
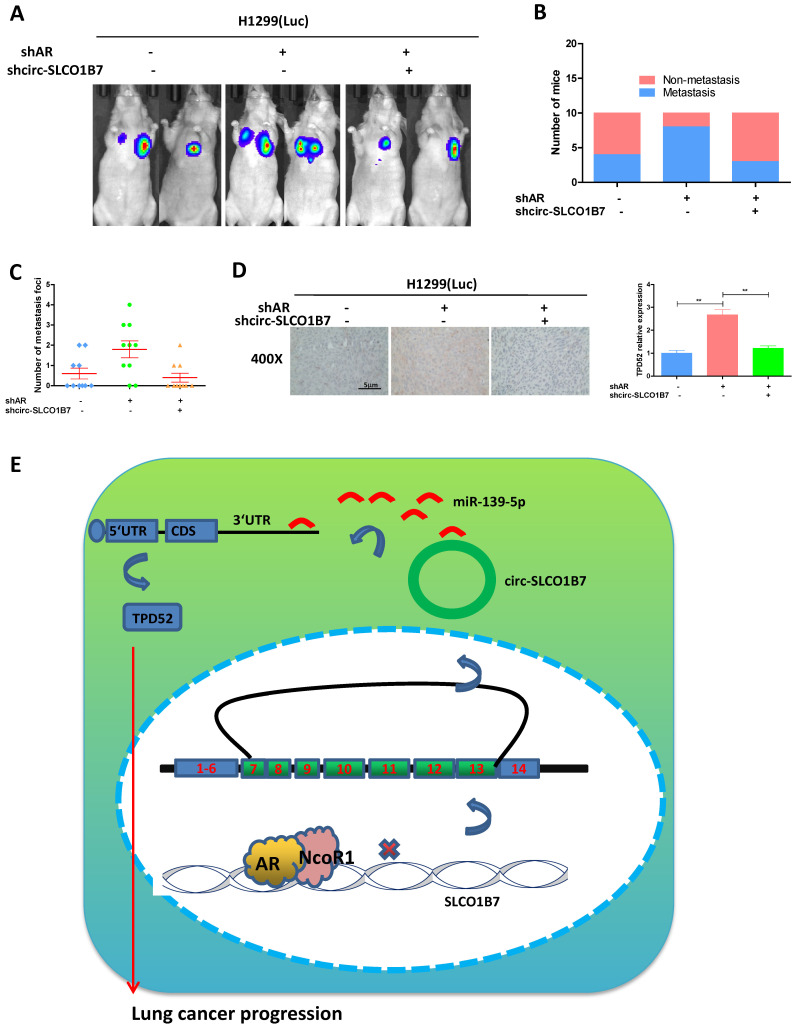
** Preclinical study using the *in vivo* mouse model to demonstrate the role of AR/circ-SLCO1B7/miR-139-5p/TPD52 signaling in the lung cancer progression.** In vivo imaging system (IVIS) imaging was performed to detect the presence of distal metastasis foci in mice. **B.** Quantification of the number of mice with metastasis. **C.** Quantification of the total number of metastatic foci. **D.** Immunohistochemistry (IHC) assay was conducted to examine TPD52 expression in tumor tissues of mice in each group. **E**. A schematic depiction of how AR regulated circ-SLCO1B7 contributed to lung cancer progression. All quantitations are presented as mean ± SD, and p-values were calculated using the t-test. Significance level is denoted as **p < 0.01.

In our preclinical study using an in vivo mouse model, we aimed to validate the mechanisms uncovered in vitro and determined their significance in lung cancer progression. To achieve this, we injected 1X10^6^ H1299-Luc cells with different gene manipulations: 1) pLKO+pLKO (control); 2) shAR+pLKO; and 3) shAR+shcirc-SLCO1B7, into the left lateral thorax of nude mice in situ. The results demonstrated that loss of AR promoted lung cancer metastasis, however, when shcirc-SLCO1B7 was introduced in combination with shAR, it effectively blocked shAR-mediated lung cancer metastasis** (Fig. 7A)**. To further confirm the metastatic foci observed from the IVIS system, we meticulously examined and quantified the number of metastatic foci after the histological staining. As expected, our quantification analyses were consistent with the IVIS signal observed earlier, demonstrating a higher number of metastatic foci in the shAR group compared to the control group, which could be attenuated in counterparts bearing lung cancer with depletion of both AR and circ-SLCO1B7** (Fig. 7B and C)**. Immunohistochemistry (IHC) analysis of TPD52 expression also demonstrated that AR/circ-SLCO1B7 axis functioned to regulate TPD52 expression (**Fig. 7D**).

Overall, AR plays an important role in suppressing lung cancer progression by altering the circ-SLCO1B7/TPD52 signaling axis (**Fig. 7E**).

## Discussion

In this study, we aimed to investigate the downstream signaling molecules responsible for the suppressive role of AR in lung cancer cell progression. We found that AR exerts its suppressive effects by transcriptionally silencing the expression of circ-SLCO1B7, a circular RNA molecule. This regulation is fulfilled via the co-recruitment of AR and NcoR1 to the 5' promoter region of SLCO1B7. The decreased expression of circ-SLCO1B7 alleviates its sponge as miR-139-5p, allowing miR-139-5p to suppress TPD52 messenger RNA and leading to the retarded lung cancer progression. Overall, these findings provide insight into the molecular mechanisms underlying the suppressive role of AR in lung cancer cell progression.

AR has been implicated in various aspects of lung cancer progression based on several reports 9, 37, 38. The role of androgen receptor (AR) signaling in tumor initiation and progression is indeed complex, with studies reporting contrasting findings. Some studies suggest that AR signaling may promote tumor initiation and early-stage development, while others propose a suppressive role in later-stage tumor progression and therapy resistance 15, 16. A previous study has reported a detrimental effect on survival in lung cancer patients receiving hormone replacement therapy 39. Our study aligns with these finding by demonstrating that the loss of androgen receptor (AR) promotes lung cancer cell invasion and decreases drug response. These results support the notion that AR acts as a tumor suppressor in late-stage tumor progression.

In our study, we made an important discovery regarding the regulatory role of circRNAs in lung cancer progression. Specifically, we found that AR-regulated circ-SLCO1B7, a circular RNA molecule, plays a significant role in lung cancer cell invasion and response to cisplatin. Numerous studies have demonstrated the involvement of non-coding RNA (ncRNA) in the development and progression of non-small cell lung cancer 33, 40, 41, and circRNAs have garnered significant attention in current research, as they have been found to play a crucial role in regulating various cellular events through interactions with miRNAs 42-44. Notably, previous studies have demonstrated the regulatory capacity of specific circRNAs in different types of cancer. For instance, Sang et al. reported that circRNA_0025202 regulates breast cancer tamoxifen sensitivity and tumor progression by modulating the miR-182-5p/FOXO3a axis 45. Similarly, Zeng et al. showed that CircHIPK3 promotes colorectal cancer growth and metastasis by acting as a sponge for miR-7 46. To extend our understanding of circRNAs, our study also illustrated that circ-SLCO1B7 exerts its suppressive effects on lung cancer by sponging miR-139-5p.

MiRNAs play critical roles in tumor development, as evidenced by several studies 15, 16, 20, 25, 47. They exert their regulatory effects by negatively modulating the expression of target genes, predominantly through binding to the 3'UTR, leading to mRNA degradation or inhibition of protein translation 48, 49. In our study, we observed that miR-139-5p specifically targets the 3'UTR of TPD52 mRNA, resulting in decreased expression of TPD52. This finding was further validated by overexpressing miR-139-5p or using a miR-139-5p inhibitor, which demonstrated consistent effects on TPD52 expression. However, our data also indicated that the reversal of AR regulated TPD52 expression by miR-139-5p was only partial, suggesting that sponging miR-139-5p is just one of the signaling pathways involved in the AR-circ-SLCO1B7-mediated lung cancer progression. It is possible that there are other signaling pathways may also interact with miR-139-5p or that additional mechanisms contribute to the observed effects, which require further investigation.

Through our analysis of the TCGA database, we observed a potential tumor-promoting role of TPD52 in lung cancer. TPD52 is an oncogene located in the amplified region of human chromosome 8q21, and its abnormal expression has been observed in various cancers 30. Among the family members (TPD52, TPD53, TPD54, and TPD55), TPD52 has been extensively studied due to its involvement in the malignancy of different cancer cells 50. Numerous studies have demonstrated the upregulation of TPD52 at both the mRNA and protein levels in several cancers, including lung cancer 31, 51, 52. In our study, we confirmed that TPD52 indeed acts as an oncogene and is regulated by the AR/circ-SLCO1B7/miR-139-5p signaling pathway, promoting lung cancer progression. These findings provide a foundation for targeting this signaling pathway to develop new drugs for more effective treatment of lung cancer.

## Supplementary Material

Supplementary figures and table.

## Figures and Tables

**Table 1:** Correlation between circ-SLCO1B7/miR-139-5p expression and clinical pathologic characteristics.

Characteristics	Case	circ-SLCO1B7		χ^2^	p	miR-139-5p		χ^2^	p
		low	high			low	high		
All cases	136	68	68			68	68		
Age (years)									
<60	82	39	43	0.491	0.483	42	40	0.123	0.726
≥60	54	29	25			26	28		
Gender									
Male	80	39	41	0.121	0.727	42	38	0.486	0.486
Female	56	29	27			26	30		
Smoking									
No	51	24	27	0.282	0.595	28	23	0.784	0.376
Yes	85	44	41			40	45		
Tumor size (cm)									
<3	81	51	30	13.463	<0.001*	35	46	3.694	0.055
≥3	55	17	38			33	22		
T grade									
T1-T2	90	58	32	22.207	<0.001*	35	55	13.140	<0.001*
T3-T4	46	10	36			33	13		
N grade									
N0-N1	92	61	31	30.237	<0.001*	34	58	19.352	<0.001*
N2-N3	44	7	37			34	10		
Differentiation									
Well	85	48	37	3.796	0.051	30	55	19.608	<0.001*
Moderate-Poor	51	20	31			38	13		

*Significant association.
