# Supplementary material for: Androgen receptor suppresses lung cancer invasion and increases cisplatin response via decreasing TPD52 expression
Source: Int J Biol Sci. 2023 Jul 16;19(12):3709–25. doi: 10.7150/ijbs.84577 (PMC10411467; doi:10.7150/ijbs.84577)

Supplementary Table1. Primers used in Real time PCR

Primer Name	Sequence (5’-3’)
hsa_circ_0008705 F	CATCAACCAGTTTGCAGCAG
hsa_circ_0008705 R	GAGGCCATAGGGTACGACAA
hsa_circ_0025583 F	TTGGGGCTCTGATTGATACA
hsa_circ_0025583 R	TCCAGACACAAGGAAACCAA
hsa_circ_0034693 F	TGAAGCTCATTTTAAGGAAATGG
hsa_circ_0034693 R	TTTTCCAGGTTTGGATGACTC
hsa-miR-1225-3p	TGAGCCCCTGTGCCGCCCCCAG
hsa-miR-1295	TTAGGCCGCAGATCTGGGTGA
hsa-miR-139-5p	TCTACAGTGCACGTGTCTCCAGT
hsa-miR-182	TTTGGCAATGGTAGAACTCACACT
hsa-miR-31	AGGCAAGATGCTGGCATAGCT
hsa-miR-485-3p	GTCATACACGGCTCTCCTCTCT
hsa-miR-516b	ATCTGGAGGTAAGAAGCACTTT
hsa-miR-556-3p	ATATTACCATTAGCTCATCTTT
hsa-miR-570	CGAAAACAGCAATTACCTTTGC
hsa-miR-635	ACTTGGGCACTGAAACAATGTCC
hsa-miR-671-5p	AGGAAGCCCTGGAGGGGCTGGAG
hsa-miR-767-3p	GGCATACCCCATGGTTTCT
hsa-miR-875-5p	TATACCTCAGTTTTATCAGGTG
COL11A1 F	ACCCTCGCATTGACCTTCC
COL11A1 R	TTTGTGCAAAATCCCGTTGTTT
MAD2L1 F	GTTCTTCTCATTCGGCATCAACA
MAD2L1 R	GAGTCCGTATTTCTGCACTCG
TOP2A F	ACCATTGCAGCCTGTAAATGA
TOP2A R	GGGCGGAGCAAAATATGTTCC
TPD52 F	AGCATCTAGCAGAGATCAAGCG
TPD52 R	AGCCAACAGACGAAAAAGCAG

Supplementary Figure legend

Figure 1 A. Chamber-transwell invasion assay was performed to assess the invasion capacity of A549 cells after overexpression of AR and circ-SLCO1B7 (oecirc-SLCO1B7). **B**. MTT assay was performed to measure the DDP response of A549 cells after overexpression of AR and circ-SLCO1B7 (oecirc-SLCO1B7). **C-D**. Conserved AR binding motif and three predicted AREs by JASPAR within the 2 kb 5'-promoter region of SLCO1B7. Significance levels are denoted as $^*p < 0.05$, $^{**}p < 0.01$, and $^{***}p < 0.001$.

Figure 2 A. Chamber-transwell invasion assay was performed to assess the invasion capacity of H1299 cells transfected with shAR or oemiR-139-5p. **B.** MTT assays were conducted to detect the DDP response of H1299 cells transfected with shAR or oemiR-139-5p. **C.** Luciferase reporter assay was performed to confirm the direct binding between miR-139-5p and circ-SLCO1B7. **D.** The expression levels of circ-SLCO1B7, miR-139-5p, TPD52 in A549 cells treated with 1 nM and 10 nM DHT. **E**. The expression levels of circ-SLCO1B7, miR-139-5p, TPD52 in H1229 cells treated with 10 μM and 20 μM enzalutamide. **F.** TPD52 stability with or without miR-139-5p. 5 μM Actinomycin D was used in this assay. $^*p < 0.05$, $^{**}p < 0.01$, and $^{***}p < 0.001$.

Figure 3 A. Survival curve of TPD52 expression in lung cancer patients from the TCGA database. **B.** AR expression in TPD52-high and TPD52-low lung cancer samples. **C**. MiR-139-5p expression in TPD52-high and TPD52-low lung cancer samples. **D.** The expression of miR-139-5p in different metastatic stage of lung cancer. Significance levels are denoted as $^*p < 0.05$, $^{**}p < 0.01$, and $^{***}p < 0.001$.

Supplementary Fig. 1

A

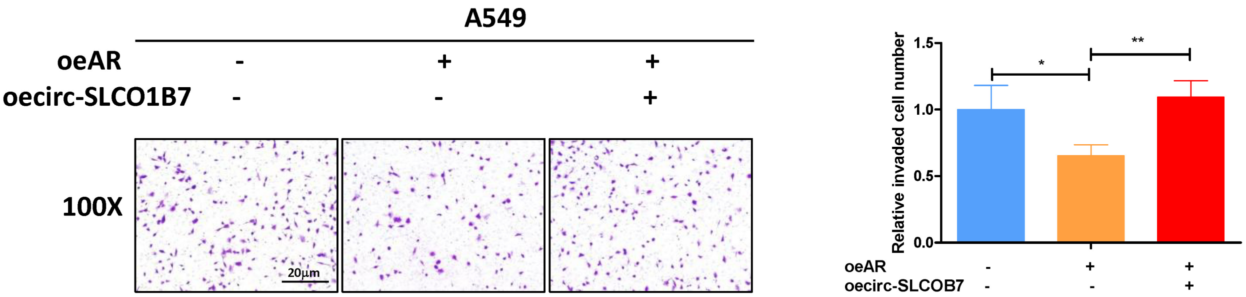

B

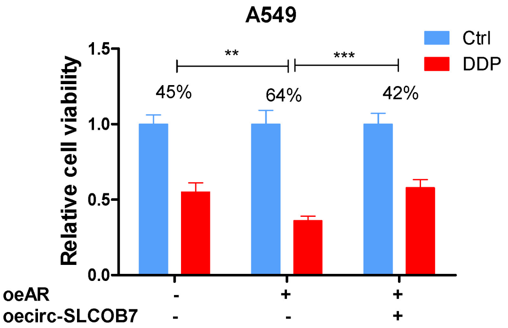

C

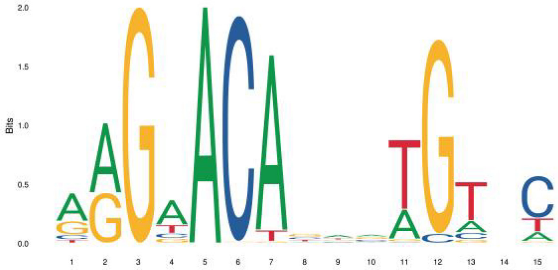

D

Score	Start	End	Strand	Predicted sequence
13.047663	1472	1486	-	AAGCACACAAAGTGT
11.211198	1468	1482	+	GAGCACACTTTGTGT
9.930478	1043	1057	-	CAGGACATGCAGTTT

Supplementary Fig. 2

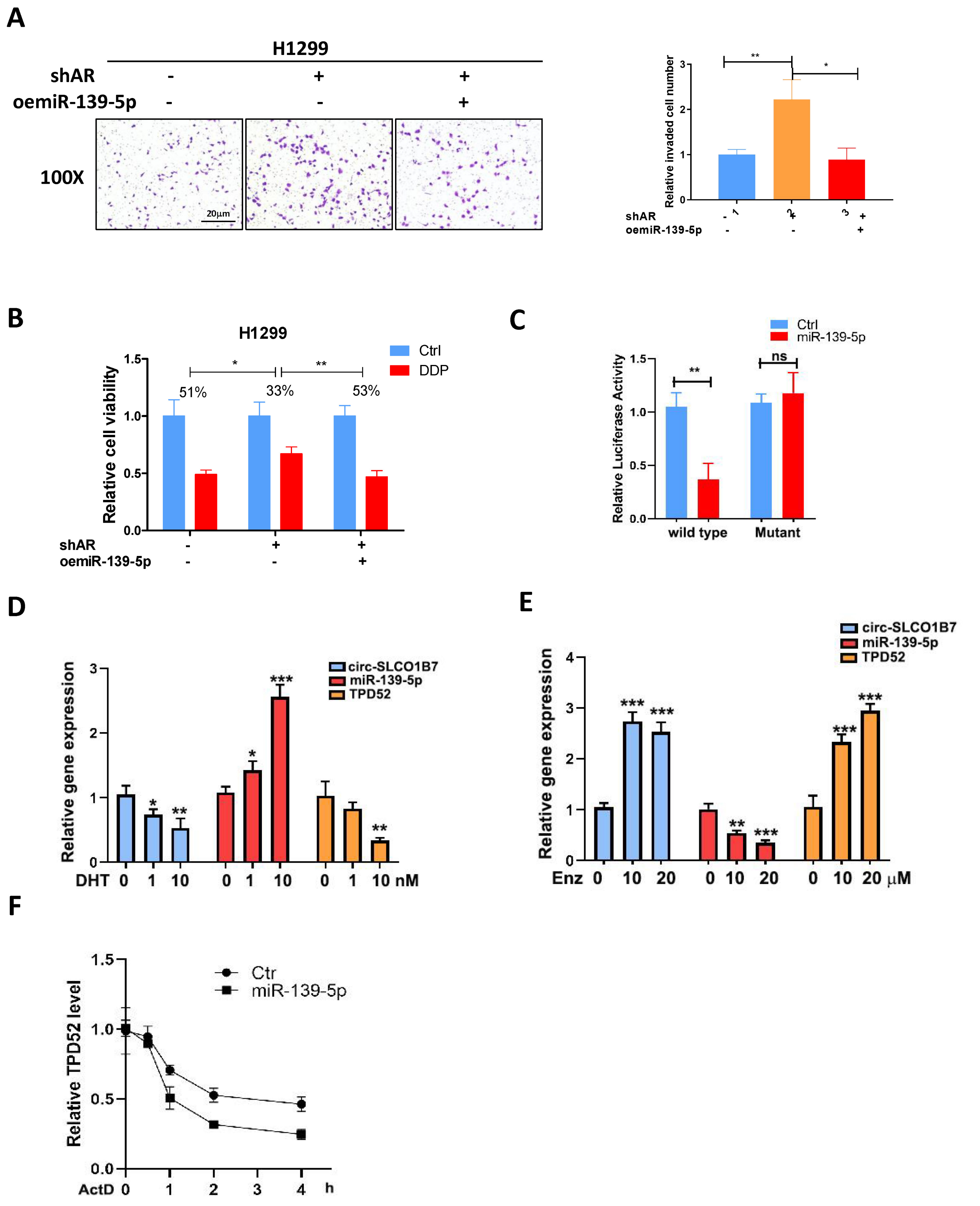

Supplementary Fig. 3

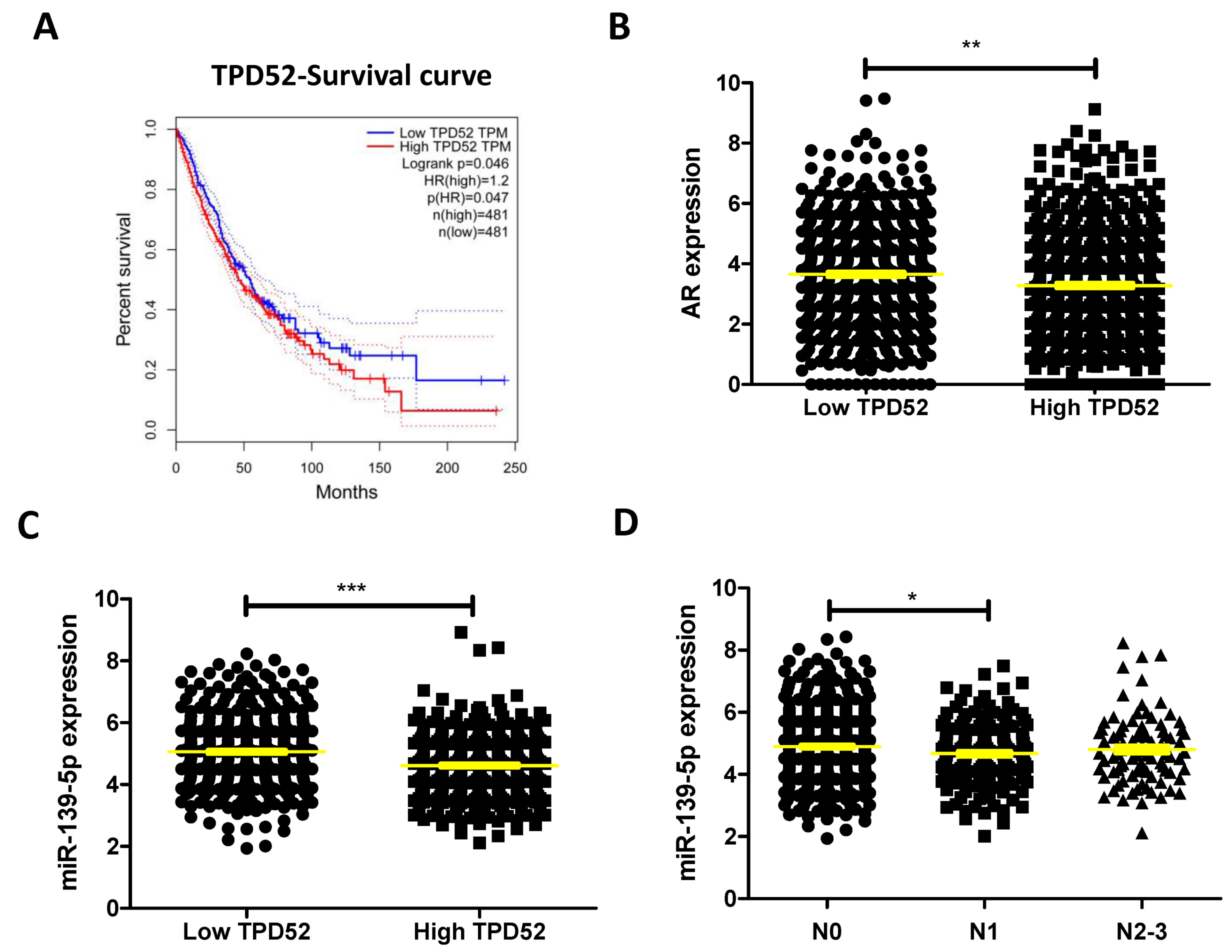

Supplement: Supplementary file 1 — Supplementary figures and table. [file ijbsv19p3709s1.pdf]
